# Salivary Levels of Titanium, Nickel, Vanadium, and Arsenic in Patients Treated with Dental Implants: A Case-Control Study

**DOI:** 10.3390/jcm9051264

**Published:** 2020-04-27

**Authors:** Piero Papi, Andrea Raco, Nicola Pranno, Bianca Di Murro, Pier Carmine Passarelli, Antonio D’Addona, Giorgio Pompa, Maurizio Barbieri

**Affiliations:** 1 Department of Oral and Maxillo-Facial Sciences, “Sapienza” University of Rome, 00161 Rome, Italy; piero.papi@uniroma1.it (P.P.); a.raco@hotmail.it (A.R.); bianca.dimurro@uniroma1.it (B.D.M.); giorgio.pompa@uniroma1.it (G.P.); 2 Division of Oral Surgery and Implantology, Department of Head and Neck, Oral Surgery, and Implantology Unit, Institute of Clinical Dentistry, Fondazione Policlinico Universitario A. Gemelli IRCCS-Università Cattolica del Sacro Cuore, 00168 Rome, Italy; piercarminepassarelli@hotmail.it (P.C.P.); antonio.daddona@policlinicogemelli.it (A.D.); 3 Department of Earth Sciences, “Sapienza” University of Rome, 00161 Rome, Italy; maurizio.barbieri@uniroma1.it

**Keywords:** dental implants, saliva, corrosion, titanium, metallic ions

## Abstract

Background: Recent articles have hypothesized a possible correlation between dental implants dissolution products and peri-implantitis. The null hypothesis tested in this case-control study was that there would be no differences in salivary concentrations of titanium (Ti), vanadium (V), nickel (Ni) and arsenic (As) ions among patients with dental implants, healthy (Group A) or affected by peri-implantitis (Group B), compared to subjects without implants and/or metallic prosthetic restorations (Group C). Methods: Inductively coupled plasma mass spectrometry was used to analyze saliva samples. One-way repeated-measure analysis of variance (ANOVA) was used to identify statistically significant differences in the salivary level of Ti, V, Ni and As between the three groups. Results: A total of 100 patients were enrolled in the study (42 males and 58 females), distributed in three groups: 50 patients in Group C, 26 patients in Group B and 24 patients Group B. In our study, concentrations of metallic ions were higher in Group A and B, compared to the control group, with the exception of vanadium. However, there were no statistically significant differences (*p* > 0.05) for metallic ions concentrations between Group A and Group B. Conclusions: Based on our results, there are no differences in titanium or other metals concentrations in saliva of patients with healthy or diseased implants.

## 1. Introduction

Dental implants are usually made of commercially pure titanium or titanium-based alloys (Ti-6Al-4V), these metals, as well as showing great long-term success and survival rates [1,2], are known to be bio compatibles and chemically inert in the oral cavity and consequently considered corrosion-resistant. Corrosion is a state of metal’s deterioration caused by oxidation or chemical action, thwarted by a titanium dioxide layer (TiO_2_), spontaneously covering the implant surface in presence of oxygen and providing great resistance and stability to the implant [3,4]. Nevertheless, titanium alloys can be affected by this gradual degradation and the structural and mechanical integrity of the implant may be jeopardized and at risk of failure. Corrosion leads to titanium dissolution, release and dispersion of metal ions and particles into soft and hard tissues either, which may be a possible factor in peri-implant inflammatory processes [5,6].

The factors that trigger corrosion are acidification of the oral environment or the release of lactic acid by bacteria, such as *Streptococcus mutans*, and also chemical stimuli by fluorides (with a concentration range greater than 100 ppm) normally present in home and professional oral care products [7,8,9].

Titanium and other metals particles released by dental implants are not totally bioinert: they induce the release of mediating inflammation cytokines, such as tumor necrosis factor-alpha (TNF-α), interleukin 1 beta (IL-1β) and the secretion of RANKL. These present an immunogenic potential which—by acting as a secondary inflammatory stimulus in peri-implantitis—amplifies bone resorption [10]

Under physiological conditions, osteoprotegerin (OPG) secreted by the fibroblasts of the periodontal ligament and osteoblasts, subtracts the receptor activator of nuclear factor kappa-Β ligand (RANKL) from the link with its true receptor activator of nuclear factor kappa-Β (RANK), creating a real competitive inhibition, preventing differentiation from the pre-osteoclast into osteoclasts and subsequent bone resorption [11,12]. In the presence of inflammation, an up-regulation of RANKL is associated with a down-regulation of OPG, which is produced in minimal quantities. The RANKL produced will, therefore, bind the RANK present on the osteoclastic precursors, with consequent massive bone resorption [13,14]

Furthermore, other kinds of corrosion can occur, such as mechanical corrosion due to functional stresses of implants or the cracking or weakening of its prosthetic components [7,8].

Once ultimately dispersed in the peri-implant environment, metal ions and particles are no longer biocompatible and inert, and are, therefore considered a potential incentive to the inflammatory process in peri-implantitis [5]. A study in mice immune cells has demonstrated that Titanium ions release pro-inflammatory cytokines involved in bone resorption, demonstrating that peri-implantitis may be directly related to corrosion and, therefore, with metal ions dispersion [15].

Peri-implantitis treatment and risk factors are still controversial [16,17], with incontrovertible evidence just for periodontitis, inadequate plaque control and lack of supportive periodontal therapy [18,19,20].

Furthermore, smoking, excess cement and other systemic conditions have all been described as potential risk indicators, however there is inconclusive evidence to draft clear conclusions [18,21,22,23,24,25,26].

Recent articles have hypothesized a possible correlation between titanium dissolution products and peri-implantitis [27,28,29] therefore, the aims of this case-control study were to evaluate salivary metallic ions dissolution in patients with dental implants, healthy or affected by peri-implantitis.

The null hypothesis tested was that there would be no differences in the salivary concentrations of titanium (Ti), vanadium (V), nickel (Ni) and arsenic (As) ions among patients with dental implants, healthy or affected by peri-implantitis, compared to subjects without implants and/or metallic prosthetic restorations.

## 2. Material and Methods

### 2.1. Study Design

To address the research purpose, the authors designed a case-control study, conducted at the Departments of Oral and Maxillo-Facial Sciences, at “Sapienza” University of Rome.

### 2.2. Study Population

From May 2018 to May 2019, all subjects who underwent previous implant surgery since January 2008 at the Oral Surgery Unit, Policlinico Umberto I, “Sapienza” University of Rome were identified and consecutively evaluated.

Each patient received detailed descriptions of the study protocol and all subjects signed an informed consent form to be included in the study population, according to the World Medical Declaration of Helsinki. The Institution Review Board of “Sapienza” approved the study (Ref. 4939/2018).

Eligible patients were divided into two groups: subjects with clinically healthy implants (Group A) and patients with peri-implantitis (Group B).

Patients in group A were enrolled in the study based on the following inclusion and exclusion criteria:

- Single crown implants functioning for >1 year
- No clinical signs of pathologies of the oral mucosa
- Age ≥ 18 years
- Implants classified as clinically healthy
- No antibiotic treatment in the previous three months
- Non-smoker
- No uncontrolled systemic diseases
- Not pregnant or breastfeeding
- No metal reconstruction, crowns or other prosthetic restorations present in the oral cavity

Patients in Group B had to meet the following inclusion and exclusion criteria:

- Single crown implants functioning for >1 year
- No clinical signs of pathologies of the oral mucosa
- Age ≥ 18 years
- Implants with a diagnosis of peri-implantitis
- No antibiotic treatment in the previous three months
- Non-smoker
- No uncontrolled systemic diseases
- Not pregnant or breastfeeding
- No metal reconstruction, crowns or other prosthetic restorations present in the oral cavity

Furthermore, a third group of patients (Group C) without dental implants, derived from a population of subjects attending the Oral Surgery Unit from May 2018 to May 2019 for wisdom tooth removal, was enrolled in accordance with the following inclusion and exclusion criteria:

- Absence of dental implants
- No metal reconstruction, crowns or other prosthetic restorations present in the oral cavity
- Age ≥ 18 years
- No clinical signs of pathologies of the oral mucosa
- No antibiotic treatment in the previous three months
- Non-smoker
- No uncontrolled systemic diseases
- Not pregnant or breastfeeding

### 2.3. Clinical Examination

Patients’ data collected included sex and age.

For each implant, the following clinical measurements were recorded at six sites per implant by using a periodontal probe (PCP-Unc 15, Hu-Friedy^®^, Chicago, IL, USA):

- Probing pocket depth (PPD) measured in millimeters
- Plaque index (PI) recorded with dichotomic values (present/absent)
- Mucosal redness recorded with dichotomic values (present/absent)
- Suppuration recorded with dichotomic values (present/absent)
- Bleeding on probing recorded with dichotomic values (present/absent)

### 2.4. Radiographic Assessment

Mesial and distal implant crestal bone levels were measured on standardized (Rinn Centratore XCP Evolution 2003, Dentsply, Rome, Italy) digital periapical X-rays for each implant using a calibrated software (SOPRO Imaging, Acteon Group, Norwich, UK). The implant length and width were used as references for calibration of measurements, with the bone level digitally evaluated by measuring the distance between the implant shoulder and the first visible bone contact on the implant.

Case definitions for epidemiological or disease surveillance studies of the 2017 World Workshop on the Classification of Periodontal and Peri-Implant Diseases and Conditions were adopted to establish the diagnosis of peri-implant diseases [30].

### 2.5. Saliva Collection

Five milliliters of saliva were collected from each patient using the unstimulated drainage method, or alternatively by leaving the saliva flow passively from the lower lip directly into designated sterile tubes.

Patients were advised not to take food or drink and to avoid oral hygiene procedures before saliva collection.

All samples were stored at a temperature of 1 °C, in order to block any biologic process still in place.

### 2.6. ICP-MS Analysis

Samples were analyzed using a Thermo Scientific XSERIES2 ICP-MS instrument (Thermo-Fisher Scientific, Waltham, MA, USA) at the Department of Earth Sciences at “Sapienza” University of Rome. Samples were processed as previously described [29]. The concentrations of Ti, V, Ni and As were quantified in μg/L.

### 2.7. Statistical Analysis

The required sample size was calculated using statistics software (GPower 3.1.9.2, Heinrich-Heine-Universität, Düsseldorf, Germany). A power analysis using the repeated measures ANOVA with three measurements, an alpha level of 0.05 and a medium effect size (f = 0.56) showed that 100 subjects would be adequate to obtain 95% power in detecting a statistical difference in the salivary level of Ti, assuming a loss to follow-up of 20% [31].

A database was created using Excel (Microsoft, Redmond, WA, USA). Descriptive statistics were calculated for each variable. The Shapiro–Wilk test was used to determine whether or not the data conformed to a normal distribution.

One-way repeated-measures ANOVA was used to identify statistically significant differences in the salivary level of Ti, V, Ni and As between three different groups: Control group, patients with healthy implant and patients with implant affected by peri-implantitis. Pairwise Tukey honestly significant difference (HSD) test for multiple comparisons.

The chi-squared test of homogeneity was used to evaluate the presence of differences in the proportion of males and females among the three groups.

Data were evaluated using standard statistical analysis software (version 20.0, Statistical Package for the Social Sciences, IBM Corporation, Armonk, NY, USA). A *p* value <0.05 was considered as statistically significant.

## 3. Results

A total of 100 patients were enrolled in the study (42 males and 58 females; age 49.02 ± 11.37 years). The patients were thus distributed in the three groups: 50 patients in Group C, 26 patients in Group B and 24 patients Group B. There were no significant differences in the proportions of males and females in the three groups (*p* = 0.620). Patients’ demographics are reported in Table 1.

### 3.1. Titanium

The mean concentration of Ti was 136.65 ± 263.28 μg/L in the Group C, 489.60 ± 227.86 μg/L in the Group A and 492.83 ± 313.90 in the Group B. Statistical significant differences in mean concentration were found between the Group C and the Group A (*p* < 0.001) and between the Group C and the Group B (*p* < 0.001) (Table 2).

### 3.2. Nickel

The mean concentration of Ni was 4.77 ± 8.33 μg/L in the Group C, 24.99 ± 12.47 μg/L in the Group A and 23.50 ± 10.12 in the Group B. Statistical significant differences in mean concentration were found between the Group C and the Group A (*p* < 0.001) and between the Group C and Group B (*p* < 0.001) (Table 2).

### 3.3. Vanadium

The mean concentration of V was 1.30 ± 4.28 μg/L in the Group C, 2.27 ± 2.43 μg/L in the Group A and 2.01 ± 1.35 in the Group B. The group means of V concentration were not statistically significant different (*p* = 0.440) (Table 2).

### 3.4. Arsenic

The mean concentration of As was 0.01 ± 0.01 μg/L in the Group C, 2.20 ± 1.88 μg/L in the Group A and 1.54 ± 2.07 in the Group B. Statistical significant differences in mean concentration were found between the Group C and the Group A (*p* < 0.001) and between the Group C and the Group B (*p* < 0.001) (Table 2).

## 4. Discussion

The aims of this study were to detect salivary metallic ions levels of Ti, V, Ni and As in patients with and without dental implants.

The metal concentration was measured by the use of ICP-MS, a versatile, rapid and extremely sensitive analytical technique used to determine different metallic and non-metallic inorganic substances present in concentrations lower than one part per billion.

Just a few studies have investigated the possible relationship of metallic ions dissolution and peri-implantitis, mainly focusing on titanium levels [27,28,29]

Safioti et al. first hypothesized a possible association between high titanium levels and implants affected by peri-implantitis, studying the metal concentration in submucosa plaque. The plaque samples were taken from the deepest points of the pockets of each implant through dental scalers (mini-five 1–2 Gracey) and then analyzed by ICP-MS. Results showed that implants with peri-implantitis had significantly (*p* < 0.05) higher titanium levels than healthy implants [27].

Olmedo et al. using the exfoliative cytology technique, measured the concentration of titanium particles in the peri-implant mucosa cells and found higher values in the group of patients affected by peri-implantitis compared to the group of patients with healthy implants [28,32].

In our study setting, attention was focused not only on titanium but also on other metals such as V, Ni and As.

Vanadium is ubiquitous: it is present in water and soil and its effects at systemic level are still debated: several authors have reported the use of vanadium in the treatment of diabetes mellitus, while others have correlated the long-term exposure with risk of cancer [33,34,35,36].

Nickel is known to be the cause of contact dermatitis and in general of allergic episodes, which can develop with serious consequences in the most sensitive subjects. Adverse reactions were documented in relation to orthodontic devices (brackets, arches) containing nickel. This element has a carcinogenic and mutagenic effects; therefore, exposure must be minimized [37,38,39].

Arsenic is one of the most widespread elements in nature: it can be found in soil, water, air and almost in all animal and plant tissues.

Arsenic poisoning can be acute (lethal) or chronic, caused by prolonged exposure even at low concentrations.

Martin-Camean et al. in 2014, in an in vivo study, evaluated the dissolution of different metal ions in the oral mucosa of subjects with orthodontic mini-implants. The epithelial cells of the oral mucosa were taken from each patient using a rubber brush. The samples were then analyzed through ICP-MS. The results reported only traces of vanadium, while the release of other elements occurred in the following growing order Cr < Ni < Ti < Cu < Al [40,41,42].

In our study, concentrations of metallic ions were higher in subjects with dental implants, either healthy or affected by peri-implantitis, compared to the control group, with the exception of vanadium.

Based on our results, metallic ions are detectable in the saliva of patients with dental implants; according to a recent systematic review conducted by Noronha Oliveira et al., degradation products—in the form of micro and nanoscale particles—can be found for different reasons, such as detachment from implant surface during surgical insertion, wear caused by micro-movements between contacting surfaces at implant connections, corrosive effects of therapeutic substances, like bleaching agents and fluorides or peri-implantitis treatment (mainly implantoplasty) [43].

In the present study, there were no statistically significant differences (*p* > 0.05) for metallic ions concentrations between patients with healthy dental implants and peri-implantitis subjects.

Our results are in contrast with the findings of Safioti et al. and Olmedo et al. [27,28]. However, in the above-mentioned articles, exclusion criteria did not limit enrollment to patients without other metallic prosthetic reconstructions and a control group without implants was not provided.

A recent systematic review by Gomes et al. [44] evaluated the diagnostic accuracy of biomarker levels in saliva to distinguish between healthy implants and implants affected by peri-implantitis. Based on their results, there was no clear evidence to support the use of salivary biomarkers (IL-6, IL-1β) in peri-implantitis detection. Pettersson et al. [45] performed gingival biopsies during surgical treatment of patients with severe periodontitis or peri-implantitis in order to evaluate titanium levels via ICP-MS. They found higher titanium values in peri-implantitis patients (*p* < 0.001) compared to periodontitis subjects, however samples were obtained after surgical treatment of dental implants and titanium release may be, therefore, exacerbated by ultrasonic scaling, as previously demonstrated by Eger et al. [46].

Furthermore, human samples from appropriate control groups (healthy implants and patients without metallic reconstructions) could not be obtained for ethical reasons.

Therefore, saliva collection and analysis through ICP-MS, as performed in the present study, seem a viable alternative to investigate data obtained by different populations.

## 5. Conclusions

Current evidence on the possible role of titanium or other metal particles in peri-implantitis pathogenesis is still controversial: in a recent critical review, Mombelli et al. concluded that there is poor specificity for the association of titanium particles and peri-implantitis, since metallic ions can be commonly detected in healthy and diseased peri-implant mucosa, as well as in gingiva of subjects without dental implants, being Tio2 used in multiple kinds of foods, toothpastes, cosmetics or medical pills [10].

Schwarz et al. in the latest World Workshop on the Classification of Periodontal and Peri-Implant Diseases and Conditions [18] stated that there was insufficient available evidence to consider titanium particles as a risk indicator for peri-implantitis.

Based on our results, concentrations of metallic ions were higher in subjects with dental implants, either healthy or affected by peri-implantitis, compared to the control group, with the exception of vanadium. No statistically significant differences were found in the metallic concentrations of healthy implants or peri-implantitis.

Future research should be orientated in conducting further studies, with a larger sample and a longitudinal design, to determine the role of titanium or other metals in peri-implantitis pathogenesis.

## Figures and Tables

**Table 1:** Sample demographic.

Study Variable	Group A	Group B	Group C
Sample size	26	24	50
Male	11	11	20
Female	15	13	30
Age (y) ± SD (range)	63.13 ± 17.72	70.52 ± 8.24	46.57 ± 9.23
Dental implants (n)	26	24	NA
Functional loading (y) ± SD (range)	7.8 ± 2.62	9.88 ± 3.52	NA
Mean PPD (mm)	3.2 ± 0.44	4.66 ± 1.32	NA
Mean MBL (mm)	0.89 ± 0.32	1.95 ± 1.43	NA

SD, standard deviation; PPD, probing pocket depth; MBL, marginal bone loss; NA, not applicable.

**Table 2:** Difference in the mean concentration of titanium, nickel vanadium and arsenic in the three groups.

Multiple Comparisons							
Tukey’s HSD							
Dependent Variable	(I) Groups	(J) Groups	Mean Difference (I-J)	Std. Error	Sig.	95% Confidence Interval	
						Lower Bound	Upper Bound
Titanium	Group C	Group A	−352.951280 *	59.517272	0.000	−494.61555	−211.28701
		Group B	−356.184613 *	61.127007	0.000	−501.68041	−210.68882
	Group A	Group C	352.951280 *	59.517272	0.000	211.28701	494.61555
		Group B	−3.233333	69.678791	0.999	−169.08426	162.61760
	Group B	Group C	356.184613 *	61.127007	0.000	210.68882	501.68041
		Group A	3.233333	69.678791	0.999	−162.61760	169.08426
Nickel	Group C	Group A	−20.215246 *	2.410887	0.000	−25.95369	−14.47680
		Group B	−18.728483 *	2.476093	0.000	−24.62213	−12.83483
	Group A	Group C	20.215246 *	2.410887	0.000	14.47680	25.95369
		Group B	1.486763	2.822503	0.858	−5.23142	8.20494
	Group B	Group C	18.728483 *	2.476093	0.000	12.83483	24.62213
		Group A	−1.486763	2.822503	0.858	−8.20494	5.23142
Vanadium	Group C	Group A	−0.965529	0.809320	0.460	−2.89189	0.96083
		Group B	−0.707260	0.831209	0.672	−2.68572	1.27120
	Group A	Group C	0.965529	0.809320	0.460	−0.96083	2.89189
		Group B	0.258269	0.947497	0.960	−1.99698	2.51352
	Group B	Group C	0.707260	0.831209	0.672	−1.27120	2.68572
		Group A	−0.258269	0.947497	0.960	−2.51352	1.99698
Arsenic	Group C	Group A	−2.193412 *	0.336286	0.000	−2.99385	−1.39298
		Group B	−1.525553 *	0.345382	0.000	−2.34764	−0.70347
	Group A	Group C	2.193412 *	0.336286	0.000	1.39298	2.99385
		Group B	0.667859	0.393701	0.212	−0.26924	1.60496
	Group B	Group C	1.525553 *	0.345382	0.000	0.70347	2.34764
		Group A	−0.667859	0.393701	0.212	−1.60496	0.26924

* The mean difference is significant at the 0.05 level. HSD, honestly significant difference.
